# Evaluation of Bone Healing on Sandblasted and Acid Etched Implants Coated with Nanocrystalline Hydroxyapatite: An *In Vivo* Study in Rabbit Femur

**DOI:** 10.1155/2014/197581

**Published:** 2014-03-02

**Authors:** Lory Melin Svanborg, Luiz Meirelles, Victoria Franke Stenport, Per Kjellin, Fredrik Currie, Martin Andersson, Ann Wennerberg

**Affiliations:** ^1^Department of Prosthodontics, Faculty of Odontology, Malmö University, 205 06 Malmö, Sweden; ^2^Department of Biomaterials/Handicap Research, Institute of Clinical Sciences, Sahlgrenska Academy, Gothenburg University, 405 30 Göteborg, Sweden; ^3^Department of Prosthodontics/Dental Material Science, Institute of Odontology, Sahlgrenska Academy, Gothenburg University, 405 30 Göteborg, Sweden; ^4^Promimic AB, 412 92 Göteborg, Sweden; ^5^Department of Chemical and Biological Engineering, Applied Surface Chemistry, Chalmers University of Technology, 412 96 Göteborg, Sweden

## Abstract

This study aimed at investigating if a coating of hydroxyapatite nanocrystals would enhance bone healing over time in trabecular bone. Sandblasted and acid etched titanium implants with and without a submicron thick coat of hydroxyapatite nanocrystals (nano-HA) were implanted in rabbit femur with healing times of 2, 4, and 9 weeks. Removal torque analyses and histological evaluations were performed. The torque analysis did not show any significant differences between the implants at any healing time. The control implant showed a tendency of more newly formed bone after 4 weeks of healing and significantly higher bone area values after 9 weeks of healing. According to the results from this present study, both control and nano-HA surfaces were biocompatible and osteoconductive. A submicron thick coating of hydroxyapatite nanocrystals deposited onto blasted and acid etched screw shaped titanium implants did not enhance bone healing, as compared to blasted and etched control implants when placed in trabecular bone.

## 1. Introduction

Dental implant treatment is today a very reliable method that provides good clinical results with success rates over 90%. Generally, lower implant survival rates have been reported in the maxilla than in the mandible, due to the difference in bone structure [1–4]. However, the optimal implant surface is yet to be developed. The current aim is to develop surfaces resulting in improved success rates in implant sites with poor bone quality and quantity. Several factors have previously been identified to be of particular importance to achieve successful osseointegration. Such factors include the surface topography, at least on a micrometer level of resolution [5]. A surface with an average surface roughness (*S*_*a*_) of approximately 1.5 *μ*m has been shown to give a stronger bone response compared to smoother (*S*_*a*_ < 1.0 *μ*m) and rougher surfaces (*S*_*a*_ > 2 *μ*m) [6]. However, research is today often aimed at evaluating the importance of nanometer-sized structures, especially in the early bone healing phase. Several *in vitro* studies have shown an increased cell response to surfaces with applied nanostructures compared to surfaces without such structures [7–15]. During the last few years *in vivo* studies have also shown promising results on bone healing to different nanostructured titanium (Ti) implant surfaces [16–18]. Further, some human studies have provided evidence of improved bone healing to Ti implants with applied nanostructures [19]. Despite this, the knowledge of the importance of nanostructures in bone healing is still limited and the significance of nanoirregularities in the clinical treatment of patients is currently unknown. According to earlier experimental and clinical studies of implants with micrometer level irregularities, plasma-sprayed hydroxyapatite (HA) coated implants have a stronger initial bone response compared to conventional titanium implants. However, long-term clinical results of the same implants have been poor. This may be explained by the plasma spraying method resulting in coats of a thickness of 50–200 *μ*m and with poor adhesion to the underlying metal [20, 21]. However, it was never investigated whether the initially positive bone response to the plasma-sprayed HA coats was due to an alleged superior biocompatibility of HA, to possible alterations in surface topography, or to a greater press fit of the thicker HA-coated implants when placed in the same sized sites as the controls. To improve the coating and minimize potential problems of coat loosening, thinner HA coats have been developed. A previous study by Svanborg et al. [22] did not support the importance of a nanocrystalline HA coat deposited on sandblasted and acid etched dental implants when placed in cortical bone. Rabbit tibia is suggested to simulate the bone of the human mandible and rabbit femur that of the human maxilla [6]. It may be that nanostructures are of benefit in trabecular bone, which do not provide satisfactory initial stability in contrast to the cortical bone site investigated in our previous study [22]. The use of blasted and acid etched dental implants has shown good clinical results [23] and it would be of interest to investigate if an added coat of nanocrystalline HA further improves the early bone healing in trabecular bone. The aim of this study was to investigate if a submicron thick coating of hydroxyapatite nanocrystals would enhance the bone healing over time, when deposited on sandblasted and acid etched screw shaped implants and placed in trabecular bone.

## 2. Material and Methods

### 2.1. Implants

The implants used in this study were threaded, sandblasted and acid etched titanium screws (grade 4) having a diameter of 3.5 mm and a length of 8.5 mm (custom made). A HA coating was applied on the test implants using a modification of the technique previously described by Kjellin and Andersson, 2006 [24]. This method creates an aqueous dispersion of nanosized HA crystals, sized ~5 nm, which are coated with amino acids. The coating of amino acids presents a positive crystal charge, which makes the crystals adhere to negatively charged surfaces, such as a titanium surface. The dispersion was applied onto the implant, and the implant was rotated in a spin-coating apparatus at 3000 rpm. The coated implant was dried in air, and a heat treatment at 550°C for 5 minutes in an oxygen rich atmosphere was done in order to sinter the HA particles onto the titanium surface and to remove the amino acids. With this method, the thickness of the resulting HA layer could be varied depending on the rotating speed. The rotating speed was set in such a way that the resulting HA layer was less than a micrometer thick, which was estimated using a Leo Ultra 55 FEG high resolution scanning electron microscope (SEM) (Carl Zeiss SMT Inc., North America). Powder X-ray diffraction (XRD) was used to determine the presence of crystalline HA structures. XRD was performed using a Bruker XRD D8 Advance (Bruker AXS, Karlsruhe, Germany) and monochromatic Cu radiation. Sandblasted and acid etched titanium implants were used as control.

### 2.2. Implant Surface Analysis

The implants were examined using SEM, operating at an acceleration voltage of 10 kV (Leo Ultra 55 FEG high resolution SEM, Carl Zeiss SMT Inc.). The magnification used was ×40 000 and the micrographs were recorded at randomly chosen areas of the implants. The surface roughness was examined using a white light interferometer (MicroXAM, Phaseshift, Arizona, USA) which is as a highly suitable technique to evaluate threaded implant surfaces [25]. An ×50 objective and a zoom factor of 0.62 were used in this study. The measured area had a size of 264 × 200 *μ*m and the vertical measuring range was 100 *μ*m. The maximal resolution of the technique is 0.3 *μ*m horizontally and 0.05 nm vertically. To be able to describe the surface topography, the roughness, the waviness and shape must be taken into consideration. The standard filter used to separate micrometer roughness from waviness, and shape is a high-pass Gaussian filter. A filter size of 50 × 50 *μ*m has been used for threaded implants. To evaluate the height deviation at the nanometer level a filter size of 1 × 1 *μ*m was used in this study, as suggested by Svanborg et al. [26]. Surfascan software (Somicronic Instrument, Lyon, France) was used to do the filtration and evaluation. This equipment provides images and numerical descriptions of the surface topography. SPIP (Image Metrology, Denmark) was used to do 3D-illustrations of the surfaces. Three implants from each group were examined. Three valleys on each implant were measured and evaluated.

For numerical description of the surface topography, four parameters were used:

  
*S*_*a*_ = the arithmetic mean of the roughness area from the mean plane;
  
*S*_ds_ = density of summits, that is, number of peaks per area unit;
  
*S*_dr_ = the ratio between the developed surface area and a flat reference area;
  
*S*_ci_ = core fluid retention index.

The parameters used represent one amplitude (*S*_*a*_), one spatial (*S*_ds_), one hybrid (*S*_dr_), and one functional (*S*_ci_) value. The functional parameter, core fluid retention index (*S*_ci_), is related to the bone biological ranking based on earlier studies on micrometer level. A low value may be related to a positive biological outcome of bone anchored implants [27]. Mathematical formulas for the parameters can be found in the literature [28].

X-ray photoelectron spectroscopy (XPS) was used for characterisation of the surface chemical compositions. XPS survey spectra were obtained using a PHI 5000C ESCA System (Perkin-Elmer Wellesley, USA). An *α* excitation source was used at 250 W with an operating angle of 45°.

### 2.3. Animals and Surgical Technique

27 adult New Zeeland rabbits were divided into 3 groups (9 animals in each) with a healing time after implant insertion of 2, 4, and 9 weeks. Before surgery the animals were anaesthezised with an intramuscular injection of fentanyl 0.3 mg/mL and fluanisone 10 mg/mL (Hypnorm Vet, Janssen, Pharmaucetica, Beerse, Belgium) at a dose of 0.5 mL per kg body weight and an intraperitoneal injection of diazepam (Stesolid Novum, Alpharma, Denmark) at a dose of 2.5 mg per animal. One mL of lidocaine (Xylocain, Astra, Sweden) was administered subcutaneously in the surgical site as analgesics and the operation was performed under aseptic conditions. One HA coated implant and one control implant was inserted into the left and right femur, respectively, therefore each animal served as its own control. The implant sites were prepared under irrigation with saline using increasing diameter of drills. Thereafter, the implant was inserted in the bone under saline irrigation. A single dose of prophylactic antibiotic sulfadoxin 200 mg/mL and trimethoprim 40 mg/mL (Borgal, Intervet, Boxmeer, Netherlands) at a dose of 0.5 mL/kg and 0.5 mL buprenorphine 0.3 mg/mL (Temgesic, Schering-Plough, Belgium) were administrated immediately after the surgery. Right after surgery the rabbits were kept in separate cages to control the wound healing. They had free access to tap water and were fed with pellets and hay. After initial healing the rabbits were allowed to run freely in a specially designed room. The three groups of animals were sacrificed after 2, 4, and 9 weeks of healing with 10 mL overdose of pentobarbital 60 mg/mL (Pentobarbital-natrium, Apoteksbolaget, Sweden).

### 2.4. Removal Torque Analysis

A removal torque analysis was performed on each implant with an electrically controlled removal torque unit. The implants were subjected only to the necessary torque (Ncm), to interrupt osseointegration, but were then not screwed out from the bone any further. This was done to enable histological evaluations of the bone complex.

### 2.5. Specimen Preparation and Histological Evaluation

**Figure 1:**
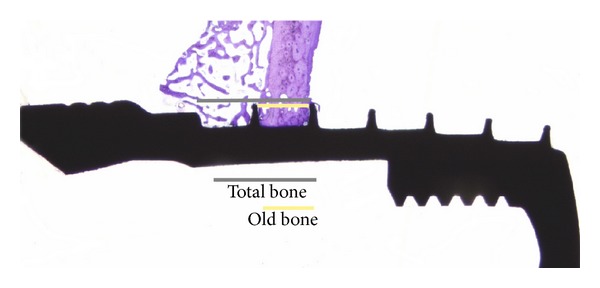
Method of measuring total bone and old bone for the new bone calculation. The new bone was calculated from the total amount of bone on each side of the implant on each histological sample minus the amount of old bone; then the percentage was calculated.

After the torque analysis, each implant was removed in a block with the surrounding bone and fixed in 4% neutral buffered formaldehyde. Then the samples were dehydrated in alcohol solutions and embedded in light curing resin (Technovit 7200 VLC, Kultzer & co, Germany). The cutting and grinding was performed as described by Donath [29]. The final sections were approximately 20 *μ*m thick and stained with toluidine-blue. Histological evaluations were performed using a light microscope together with an image analysis software (Image analysis 2000, Sweden). The evaluations included measurements of the amount of new bone (NB) and bone area (BA) along the entire implant. The amount of NB was calculated from the total amount of bone minus the amount of old bone (Figure 1) with a ×4 objective and a ×10 lens when needed for visualization. The bone area (BA) was evaluated in each thread on each implant and on the upper threadless part of the implant. The evaluations were made using a ×10 objective and were presented as the mean value of all threads on the entire implant and as a mean of the three best threads on each side of the implant on histological sample. All measurements were made using a ×10 eye-pice and in a blinded manner.

### 2.6. Statistics

The statistical analysis was performed using SPSS (statistical package for the social studies). Mann-Whitney *U*-test was used and differences were considered significant at *P* ≤ 0.05.

## 3. Results

Five animals experienced tibia fracture, two in each group with 2 and 9 weeks of healing time, and one in the group with 4 weeks of healing time. These five rabbits had to be sacrificed in advance and were not included in the results. The postoperative period was uncomplicated for the rest of the rabbits. No signs of infection or inflammation were registered at the time of implant retrieval nor were other deviations from normal observed. All implants were stable at the time of retrieval.

### 3.1. Implant Surface Characterization

**Figure 2:**
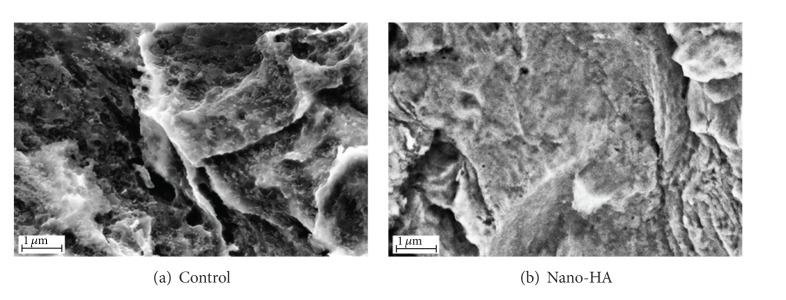
SEM images taken at ×40 000 magnification on (a) the control surface and (b) the nano-HA surface.

SEM images of the surfaces are shown in Figure 2.

**Figure 3:**
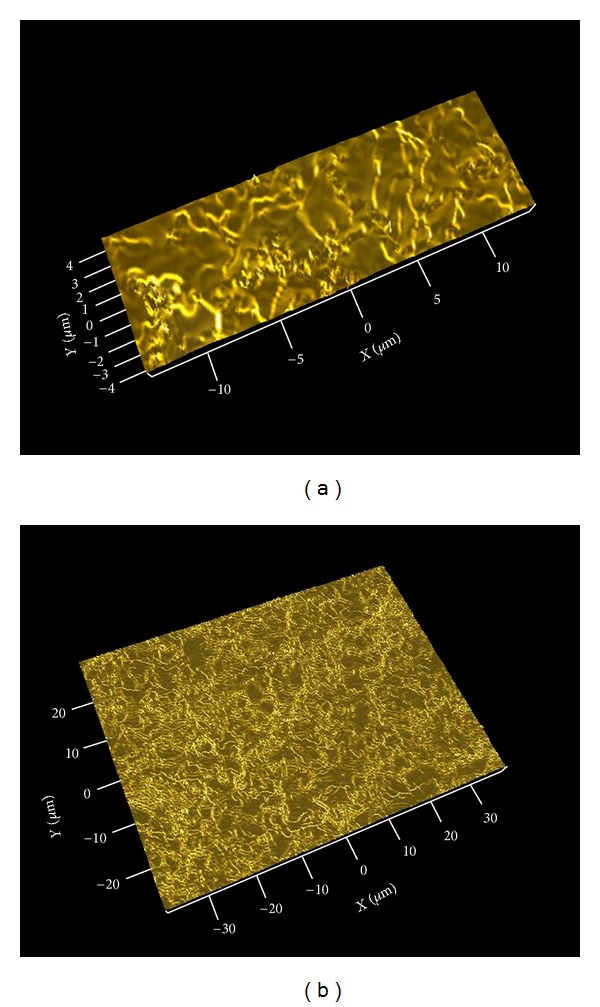
Images from the interferometer analysis of (a) the control titanium surface and (b) the test nano-HA surface.

Results from the interferometry analysis are presented in Table 1 and images of the surface topography are shown in Figure 3. Both implant types presented similar surface roughness on both micrometer and nanometer level. The mean *S*_*a*_ value on the micrometer level was 1.08 *μ*m for the control implant and 0.93 *μ*m for the nano-HA coated one. There was no significant difference (*P* > 0.05) with respect to the evaluated surface parameters between test and control implants. Further, on the nanometer level, the mean *S*_*a*_ value was 114 nm for the control and 119 nm for the nano-HA coated test implants, no significant difference (*P* > 0.05).

**Figure 4:**
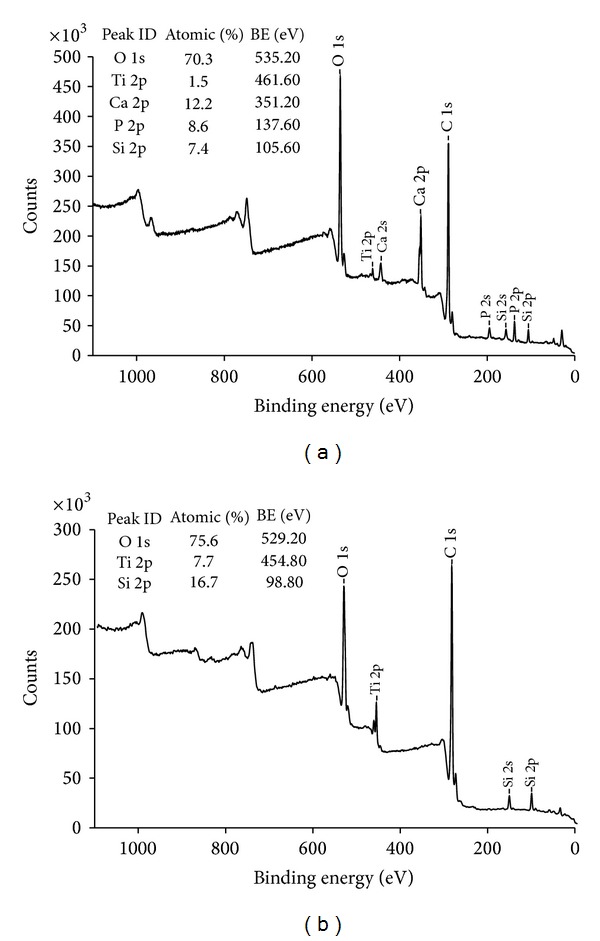
XPS survey spectra of (a) the test nano-HA surface and (b) the control titanium surface.

**Figure 5:**
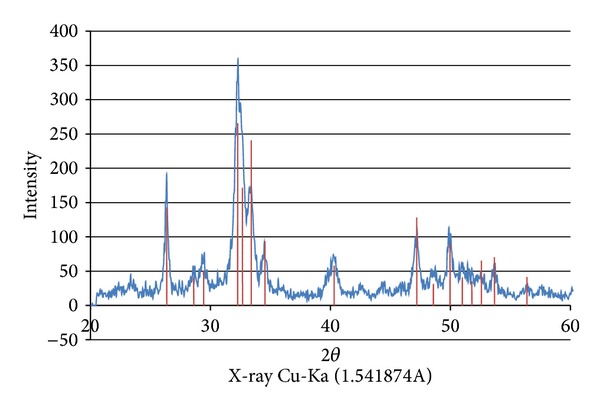
XRD demonstrating the presence of crystalline HA.

The XPS analysis showed presence of calcium and phosphorus on the surface of the coated test implants, while the controls had no such elements present (Figure 4). Furthermore, the XRD demonstrated the presence of crystalline HA (Figure 5).

### 3.2. Removal Torque Analysis

**Figure 6:**
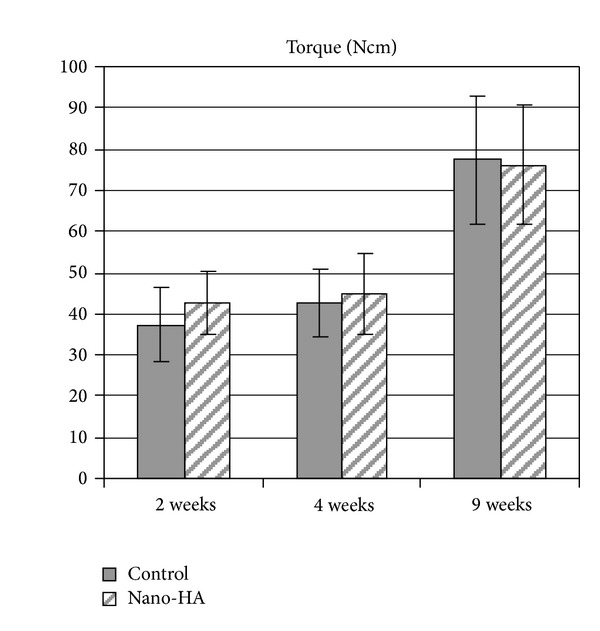
Removal torque results (mean value) after 2, 4, and 9 weeks of healing. The bar presents the standard deviation. Seven samples were evaluated in each group.

Results from the torque analysis showed no significant differences between the implant groups at any healing time (*P* > 0.05), see Figure 6. A slightly higher mean value for the nano-HA coated implants could be noted after 2 weeks of healing. No increase in torque value were seen after 4 weeks; however, after 9 weeks of healing the value increased for both implant types, but there were no significant differences between the implants.

### 3.3. Histological Results

**Figure 7:**
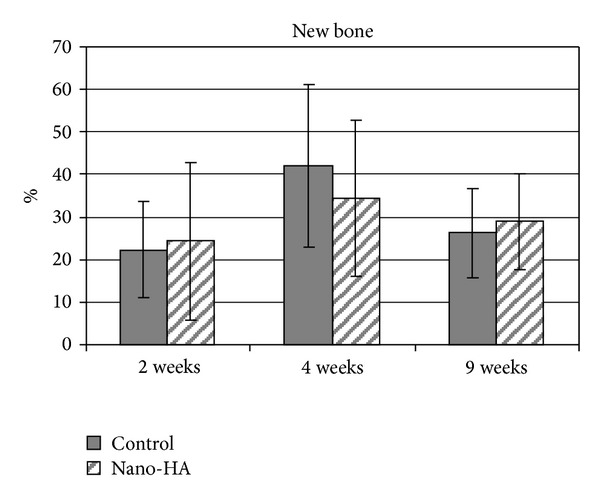
Amount of new bone after 2, 4, and 9 weeks of healing. The bar presents the standard deviation. Seven samples were evaluated in each group.

Qualitative analysis of all the samples showed a normal inflammatory response in terms of few macrophages and neutrophils observed in the histological samples. After 4 weeks of healing there was a tendency for more new bone on the control implants compared with the coated nano-HA. However, there were no significant differences at any of the chosen healing times (Figure 7).

**Figure 8:**
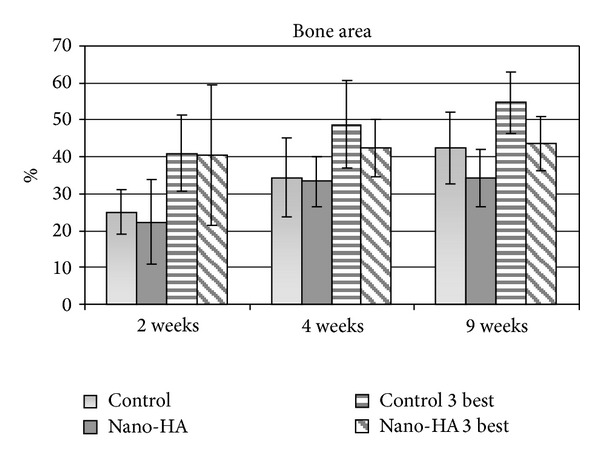
Bone area after 2, 4, and 9 weeks of healing. Presented in percentage as a mean of all threads and calculated for the three best threads on each side of each implant. The bar represents the standard deviation.

There was no difference between the implant groups when evaluating the bone area along the entire implant (Figure 8). However, when calculating the 3 best threads on each side of the samples, the control implant had a significantly (*P* = 0.025) higher value than the nano-HA after 9 weeks of healing. When evaluating the BA on the upper nonthreaded part of the implants there was also a significantly (*P* = 0.003) higher value for the control implant.

## 4. Discussion

An error search was made after the experimental part of the study was finished, since 5 rabbits unfortunately suffered from tibia fracture. The animal operations were made according to standard protocol and after well-documented procedures with no complications and by an experienced operator. After a close and strict error analysis, the authors could not find any explanation other than chance for these fractures.

The results from this study showed that both control and nano-Ha surfaces were biocompatible and osteoconductive. However, the submicron thick nano-HA coating did not improve the early bone healing compared to the control and the results support the following studies.

Coelho et al. (2009) showed that 20–50 nm thick CaP based coating on a blasted and etched cylindrical implants did not improve the biomechanical fixation or BIC after 2 and 4 weeks of healing in dog tibia [30]; an *in vivo* study in goat, on screw shaped grit-blasted, acid etched (GAE) and electrosprayed CaP nanoparticle-coated implants gave similar bone responses and torque values as to GAE alone [31]; Schliephake et al. (2009) did not find any significant difference in host response (foxhound) to dual acid etched (DAE) screw shaped implants coated with HA compared to DAE alone [32]. Further, Lee et al. (2009) concluded that screw shaped titanium or ceramic implants coated with HA nanocrystals did not improve the early bone response in rabbit [33]; Svanborg et al. (2011) confirmed similar results [22]. However, there are other *in vivo* studies having shown positive effects on bone healing to various nanostructured Ti implants [16, 18, 19, 34].

As mentioned before, one theory behind this discrepancy in reported results has been that nanostructured Ti implants may be of benefit in bone with poor quality but of insignificant importance in the healing in sites that already provide excellent initial implant stability. However, the present study of implants placed in trabecular bone did not support this theory on trabecular bone influence on implant outcome. Several *in vivo* and some clinical studies have tried to clarify the importance of nanosized structures in early bone healing and osseointegration. Although the studies are performed in different animals, the size, shape, and chemical composition of the nanostructures are also often different and therefore the studies are very difficult to compare. The difference in results from previous studies might be explained by differences in nanotopography; however the possible effect of the surface chemical composition cannot be excluded. Hence, further studies are needed to be able to conclude if some type of nanostructure may influence the bone healing and also if they might be of significance in the treatment of patients.

## 5. Conclusion

According to the results from this present study, both control and nano-Ha surfaces were biocompatible and osteoconductive. A coating of hydroxyapatite nanocrystals deposited onto blasted and acid etched screw shaped titanium implants did not enhance bone healing after 2, 4, or 9 weeks compared to a blasted and etched control implants.

## Figures and Tables

**Table 1:** Results from the interferometer characterization. The numbers represent the mean value of each parameter (the standard deviation is presented within parenthesis).

	Gauss filter 50 × 50 µm				Gauss filter 1 × 1 µm			
	*S*_*a*_ (µm)	*S*_ds_ (/mm^2^)	*S*_dr_ (%)	*S*_ci_	*S*_*a*_ (nm)	*S*_ds_ (/mm^2^)	*S*_dr_ (%)	*S*_ci_
Control	1.08 (0.41)	1 184807 (244569)	142.5 (73.0)	1.21 (0.18)	114 (11.1)	2 055650 (106081)	74.9 (13.4)	0.95 (0.12)
Nano-HA	0.93 (0.25)	1 259841 (143100)	146.5 (46.0)	1.12 (0.25)	119 (6.9)	2 132025 (78489)	83.9 (8.9)	0.84 (0.04)

*S*_*a*_: the arithmetic mean of the roughness area from the mean plane; *S*_ds_: density of summits, that is, number of peaks per area unit; *S*_dr_: the ratio between the developed surface area and a flat reference area; *S*_ci_: core fluid retention index.
